# Association Between Low Serum Creatinine and Mortality in Patients With Severe Chronic Obstructive Pulmonary Disease

**DOI:** 10.7759/cureus.29376

**Published:** 2022-09-20

**Authors:** Abdul B Afzal, Saifudin Khalid, Saumitra Baksi

**Affiliations:** 1 Respiratory Medicine, East Lancashire Hospitals NHS Trust, Blackburn, GBR

**Keywords:** copd, chronic obstructive pulmonary disease, non-invasive ventilation, prognostic factors in copd, bmi, creatinine, copd mortality

## Abstract

Introduction: Muscle mass may be a better predictor of mortality than BMI in chronic obstructive pulmonary disease (COPD). Serum creatinine depends on muscle mass and renal function; low values may predict higher mortality.

Objective: To determine whether there is an association between low serum creatinine and mortality in severe COPD.

Methods: This is a retrospective study of serum creatinine values at admission and within the last year before admission. Outcomes measured were mortality at 30 days and one year after admission in patients with acute type 2 respiratory failure secondary to COPD, who were admitted over a one-year period to a respiratory ward (N = 130). The statistics were calculated using the chi-squared test.

Results: There appears to be a significant relationship between the one-year pre-admission creatinine values and mortality at one year (p = 0.0003).

Conclusions: The relationship with mortality appears to be stronger with pre-admission creatinine values rather than the admission values and appears to predict the patients at the highest risk of mortality one year after admission.

## Introduction

In chronic obstructive pulmonary disease (COPD), a low body mass index (BMI) is an adverse prognostic factor and is associated with an increase in mortality [1-4]. A composite score consisting of BMI, airflow obstruction, dyspnoea, and exercise tolerance (BODE index) is often used for prognostication in patients with stable COPD and is considered to be superior to the forced expiratory volume in one second (FEV1) alone for this purpose [5]. For prognostication during an acute exacerbation of COPD, a composite score of comorbidities including airflow obstruction, dyspnoea, exercise tolerance, and details of the severity of previous exacerbations (CODEX) was found to be superior to both the BODE index on its own or when the BODE index was used in conjunction with additional information about previous exacerbations (BODEX) [6].

Mid-arm muscle area was shown in a study to be a better prognostic indicator than BMI in patients with COPD [7]. The relationship between BMI and fat-free muscle mass index (FFMI) is not very good and FFMI is shown to give additional prognostic information in patients with COPD [4].

Serum creatinine values depend in part on muscle mass and are commonly measured as part of the routine assessment in patients admitted to the hospital with different conditions, including an exacerbation of COPD. These values are also measured quite frequently in primary care, as part of chronic disease monitoring. A previous study in unselected patients admitted to intensive care has shown both high and low serum creatinine values to be associated with increased mortality risk [8].

The DECAF (dyspnoea, eosinopenia, consolidation, acidemia, and atrial fibrillation) study on factors that predict hospital mortality in patients with COPD exacerbation included baseline serum biochemistry at the time of admission but did not include historical pre-admission creatinine values [9]. As creatinine values can also rise acutely, due to many other conditions such as the presence of renal impairment or infection, the values measured at the time of admission may be less reliably correlated to the muscle mass in an individual and this may explain why in the DECAF study a low creatinine value was not identified as a risk factor for mortality. If this is the case, it may be that the creatinine values measured during periods of stability, such as samples taken routinely in primary care, may have a better correlation with the muscle mass in an individual patient.

We postulate that low serum creatinine values either at the time of admission or before admission may be associated with an increased risk of mortality in patients with severe COPD. If this is found to be the case, it would offer the advantage of the ready availability of a prognostic indicator, as this test is frequently performed both in the primary care and secondary care setting. If so, it may help to identify patients at risk of adverse outcomes promptly.

Patients who receive non-invasive ventilation (NIV) for acute type 2 respiratory failure (commonly secondary to COPD) are at high risk of dying with an inpatient mortality rate of 34% in the British Thoracic Society national audit on NIV, 2013. We wanted to see if there would be a significant difference in mortality in this group of patients based on their admission or pre-admission creatinine levels.

## Materials and methods

The study was approved by the Health Research Authority, Health and Care Research Wales (IRAS project ID: 254977), and by the Hospital Research and Development department. We undertook a retrospective observational cohort study in patients with severe COPD, who were admitted with acute type 2 respiratory failure and treated with NIV. All patients who had received NIV for the treatment of acute type 2 respiratory failure secondary to COPD in a respiratory ward in Royal Blackburn Teaching Hospital (Lancashire, United Kingdom) in 2013 were included in the study. The diagnosis of COPD was confirmed by a review of the discharge summaries, clinic letters, or patient notes, and any patients where NIV was used for indications other than COPD (such as obesity hypoventilation or neuromuscular disease resulting in type 2 respiratory failure) were excluded. The study was performed in 2019 and the reason for selecting the cohort from 2013 was to ensure complete data capture to evaluate one-year mortality after discharge.

A total of 130 patients met the criteria for inclusion in the study. The normal reference ranges set by the hospital laboratory for creatinine were 46-92 μmol/L for women and 58-110 μmol/L for men. For the study, a patient was considered to have low serum creatinine values if they had creatinine values of 45 or lower for females and 57 or lower for males.

Mortality rates were compared at 30 days and one year after discharge between patients who had low creatinine values within the year prior to discharge and patients who had normal or high creatinine values. The first sample taken at the time of admission for serum biochemistry was taken as the admission creatinine value and any value of creatinine that was abnormally low between the preceding year and time of admission was taken as evidence of low pre-admission creatinine value for the purpose of our calculations.

## Results

We performed a chi-squared test to see if there were statistically significant differences in mortality rates observed in patients with low admission or baseline creatinine values, compared to patients with normal values, at 30 days and one year after discharge.

Patients included in the study were 35% male (N = 46) with an average age of 68.9 years (median = 69 years). The median pH of blood gases at the time of initiation of NIV was 7.29 (range = 7.19-7.34). The results are summarized in Table 1.

**Table 1:** Mortality differences between patients with low admission or pre-admission creatinine values when compared to others at 30 days and one-year post-admission

	Died within 30 days	Alive at 30 days	P-value	Died within 1 year	Alive at 1 year	P-value
Low admission creatinine (N = 31)	7	24	0.107	17	14	0.068
Normal or high admission creatinine (N = 99)	11	88		36	63	
Low creatinine within 1 year prior to admission (N = 48)	6	42	0.734	30	18	0.0003
Normal or high creatinine in the year before admission (N = 82)	12	70		25	57	

**Figure 1:**
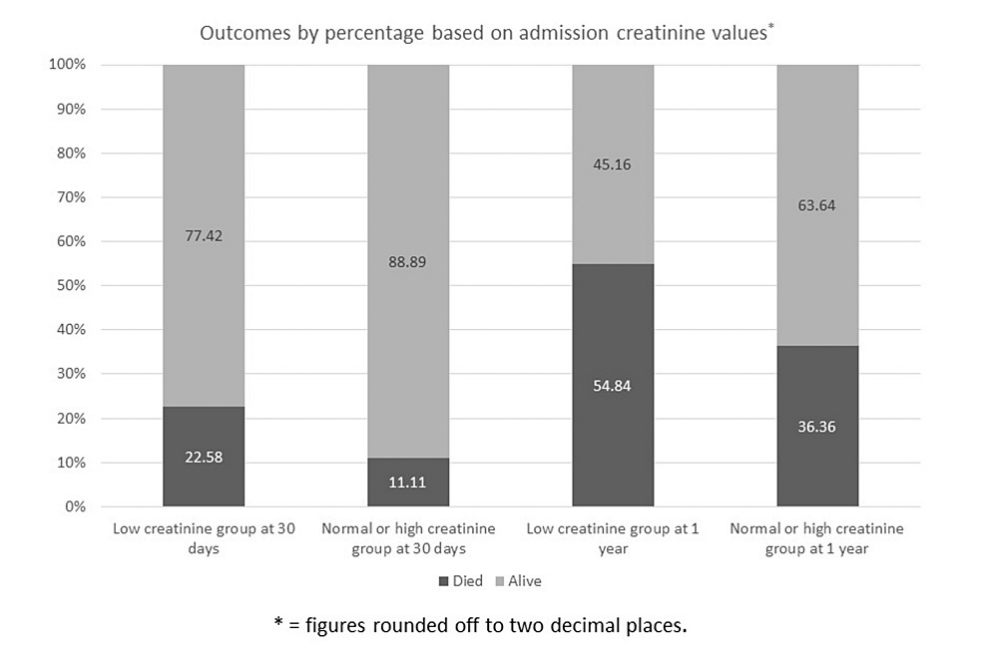
Mortality at 30 days and one year based on admission creatinine values

Based on the admission serum biochemistry, 31 patients (23.85%) out of the total of 130 patients in the study had low serum creatinine values. The mortality rate in this group was 22.58% (N = 7) at 30 days and 54.84% (N = 17) at one year (Figure 1). The differences did not reach statistical significance (p-values of 0.107 and 0.068 for 30 days and one year, respectively) when compared to patients with normal or high creatinine values based on admission values where the 30-day mortality was 11.11% (N = 11) or 36.36% at one year (N = 36).

**Figure 2:**
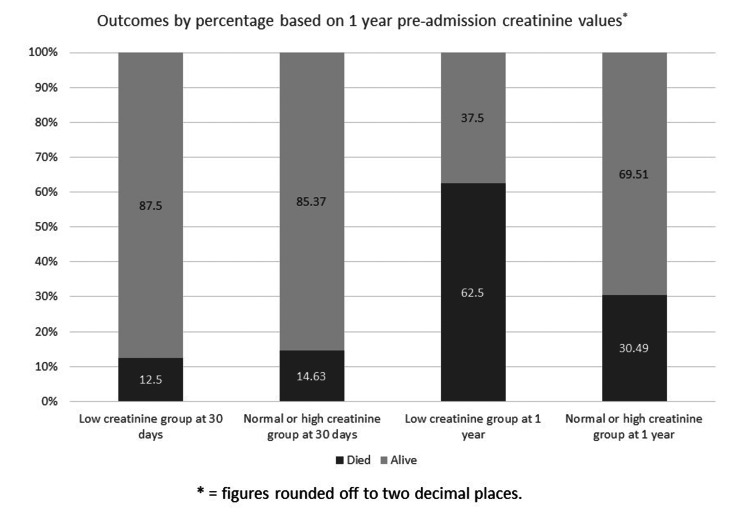
Mortality at 30 days and one year based on pre-admission creatinine values

When patients were grouped based on the presence of low creatinine values on blood tests done within one year before admission, the mortality rate in patients at 30 days in those with low creatinine (N = 6) was 12.5%, compared to 14.63% in others (p = 0.734). The mortality rate at one year, however, was significantly higher (p = 0.0003) in patients with low pre-admission creatinine where 62.5% of patients (N = 30) had died compared to 30.49% mortality (N =25) in patients with normal or high creatinine values (Figure 2).

## Discussion

Low BMI has been known to be an adverse prognostic indicator in patients with COPD, but muscle mass may be superior to BMI in this respect [7] or to show additional information than BMI [4]. Measurement of fat-free muscle mass or mid-arm muscle area is complex. In studies, this has been calculated by using equations incorporating various other factors such as mid-arm circumference, BMI, skinfold thickness, and estimation of fat mass [7]. This complexity makes the use of endpoints such as these problematic in day-to-day utilization in the care of patients with COPD. In addition to this, although there is a suggested method of categorizing cut-offs for low fat-free muscle index [10], there is no official guidance on this subject.

Serum creatinine values are routinely checked during hospital admissions and frequently measured in primary care. Although the values of serum creatinine can be influenced by a variety of factors such as intrinsic renal disease, use of nephrotoxic medications, and presence of systemic illness, values also depend on underlying muscle mass [11]. As some of these factors are more likely to be present during the period of hospitalization, it would be expected that measurement of serum creatinine values during stable conditions, perhaps in primary care, would be more closely related to the actual muscle mass than those which are measured during periods of hospitalization, as the latter ones may be falsely elevated by factors such as acute kidney injury or use of nephrotoxic drugs. This may explain why in the DECAF study low serum creatinine was not noted to be a significant factor that influenced mortality, as only the values on admission were considered.

In our study, we find that although there were no significant differences in mortality between patients with low creatinine values and others at 30 days post-admission, there appeared to be a trend toward significance at one year when patients were identified based on the admission values, and a highly significant difference when the patients were identified based on pre-admission creatinine values. This suggests that the differences in mortality may be apparent on a medium to long-term basis rather than in the immediate post-admission period. Previous studies have shown that an increase in BMI through nutritional support improved survival [3] and quality of life in patients with COPD [12]. If further studies (ideally done prospectively) confirm the value of low serum creatinine as a useful prognostic marker in identifying patients at increased risk of death, it may be possible to improve outcomes by offering nutritional support to these patients, especially as the increase in mortality is not in the immediate post-discharge period.

## Conclusions

The identification of a greater number of patients with low serum creatinine based on pre-admission values rather than the admission ones appears to suggest that some of these patients did develop an increase in their creatinine values, which might reflect acute deterioration in their overall health. This may explain the lack of significant differences in mortality based on admission values is in part due to the death of these patients in the low creatinine comparative group.

It would be recommended to confirm the findings of our study by performing a prospective study where other important factors such as the patient’s BMI, nutritional status, and severity of airflow obstruction can also be considered to assess the differences in mortality.
